# Association of Maternal Microbiota and Diet in Cord Blood Cytokine and Immunoglobulin Profiles

**DOI:** 10.3390/ijms22041778

**Published:** 2021-02-10

**Authors:** Karla Rio-Aige, Ignasi Azagra-Boronat, Malén Massot-Cladera, Marta Selma-Royo, Anna Parra-Llorca, Sonia González, Izaskun García-Mantrana, Margarida Castell, María J. Rodríguez-Lagunas, María Carmen Collado, Francisco José Pérez Cano

**Affiliations:** 1 Physiology Section, Department of Biochemistry and Physiology, Faculty of Pharmacy and Food Science, University of Barcelona (UB), 08028 Barcelona, Spain; rioaigekarla@ub.edu (K.R.-A.); ignasiazagra@ub.edu (I.A.-B.); malen.massot@ub.edu (M.M.-C.); margaridacastell@ub.edu (M.C.); franciscoperez@ub.edu (F.J.P.C.); 2 Nutrition and Food Safety Research Institute (INSA-UB), 08921 Santa Coloma de Gramenet, Spain; 3 Institute of Agrochemistry and Food Technology (IATA-CSIC), National Research Council, 46980 Valencia, Spain; mselma@iata.csic.es (M.S.-R.); igama@iata.csic.es (I.G.-M.); mcolam@iata.csic.es (M.C.C.); 4 Neonatal Research Group, Health Research Institute La Fe, 46026 Valencia, Spain; annaparrallorca@gmail.com; 5 Department of Functional Biology, Faculty of Medicine, University of Oviedo, 33006 Oviedo, Spain; soniagsolares@uniovi.es; 6 Diet, Microbiota and Health Group, Instituto de Investigación Sanitaria del Principado de Asturias (DIMISA, ISPA), 33011 Oviedo, Spain

**Keywords:** breast milk, cytokine, immunoglobulin, diet, enterotype, microbiota, cord blood

## Abstract

Mothers confer natural passive immunization to their infants through the transplacental pathway during the gestation period. The objective of the present study was to establish at birth the maternal and cord plasma concentration and relationship of immunoglobulins (Igs), cytokines (CKs), and adipokines. In addition, the impact of the maternal microbiota and diet was explored. The plasma profile of these components was different between mothers and babies, with the levels of many CKs, IgM, IgG2a, IgE, IgA, and leptin significantly higher in mothers than in the cord sample. Moreover, the total Igs, all IgG subtypes, IgE, and the Th1/Th2 ratio positively correlated in the mother–infant pair. Maternal dietary components such as monounsaturated fatty acids-polyunsaturated fatty acids and fiber were positively associated with some immune factors such as IgA in cord samples. The microbiota composition clustering also influenced the plasma profile of some factors (i.e., many CKs, some Ig, and adiponectin). In conclusion, we have established the concentration of these immunomodulatory factors in the maternal–neonatal pair at birth, some positive associations, and the influence of maternal diet and the microbiota composition, suggesting that the immune status during pregnancy, in terms of CKs and Igs levels, can influence the immune status of the infant at birth.

## 1. Introduction

Maternal–fetal coordinated communications are required to promote healthy pregnancy outcomes. In addition, mothers confer natural and passive protection to their offspring through the transplacental pathway during gestation and later by lactation [1,2]. Cytokines (CKs), immunoglobulins (Igs), and adipokines are important components in fetal development and physiology during gestation [3,4,5,6,7,8]. However, the concentrations of these factors are different between populations, ethnicities, and genetic backgrounds. To date, Kee Thai Yeo and colleagues described that Kenyan neonates had higher levels of some of these factors, such as TNF-α, than United States neonates [9].

The network of communication of the immune system is essential to maintain a tolerant and anti-inflammatory environment during pregnancy and, in turn, to protect the fetus from infections [4]. However, in the parturition an inflammatory environment is needed [3,10] to lead to correct delivery [11].

Maternal infections during birth produce higher levels of pro-inflammatory CKs in neonates [9]. These concentration changes are very important since it has been observed that the concentrations of interleukin (IL)-6, IL-10, and C-X-C Motif Chemokine Ligand 8 (CXCL8) are associated with a higher risk of being small for gestational age (SGA) and higher concentrations of tumor necrosis factor receptor 1 (TNFRI), IL-5, IL-1, IL-8, and TNF-α are related to prematurity risk [12,13,14].

Igs are glycoproteins that are divided in five classes (IgM, IgD, IgG, IgA, and IgE) according to their structure and functions [15]. Neonates have low levels of IgM and even lower levels of IgA and IgE compared to later stages of life [5]. Besides, IgG is mainly of maternal origin [5], since it is the only Ig that can be transplacentally transferred.

With regard to adipokines, the adiponectin is a pleiotropic molecule that presents anti-inflammatory, anti-fibrotic, anti-apoptotic, and proangiogenic effects, in addition to its ability to increase the sensibility to insulin, which is its most known action [16]. Recent studies have shown that the levels of this adipokine in cord blood are correlated positively with the accumulation of neonatal fat mass (FM) [17], body mass index (BMI), and birth weight [6], which is contrary to what it is observed in adults. Concerning leptin, there are controversial studies about pregnancy. On the one hand, it is reported that leptin levels increase in women during gestation because of their beneficial effects on the development of the fetal organs [7]. On the other hand, it is thought that leptin concentration in breast milk and in fetal plasma can be associated with the risk of obesity later in life, and are also linked to maternal weight [8,18].

With regard to the maternal diet, Ellen L. Mozurkewich and colleagues demonstrated that supplements of omega-3 fatty acids (eicosapentaenoic acid, EPA; docosahexaenoic acid, DHA) in pregnant women decrease levels of proinflammatory CKs and are associated positively with those of IL-10 (anti-inflammatory CK) in maternal plasma but not in cord blood [19]. In addition, during the gestational period the intake of potential food allergens induces tolerance in offspring in humans [20]. Thus, this indicates that maternal diet can influence a baby’s health during pregnancy, although the exact mechanisms are rather unknown. A possible mechanism could be the influence on the maternal microbiota which, in turn, may influence the development of the neonate. In fact, there are several studies that demonstrate the impact of the diet on the maternal [21,22,23,24,25] and infant microbiota [26,27,28].

In this study, we first aimed to characterize maternal–neonatal plasma immune factor levels and their transmission in a Mediterranean birth cohort study [29]. In a previous study with this cohort, it was determined that mothers with different diets had different intestinal microbiota compositions [28], thus we further aimed as a secondary objective at evaluating the influence of the maternal diet and gut microbiota enterotype in this passive transmission line of immunobioactive compounds.

## 2. Results

### 2.1. Demographic Data and Maternal Microbial Enterotypes

The present study was performed in a subgroup within the MAMI birth cohort (characteristics are described in Table 1). The maternal gut microbial enterotype assessed in a previous study [28] was used to determine how the gut microbial community influenced both the maternal plasma (MP) and umbilical cord plasma (UCP) immune compositions (Supplementary Figure S1). The maternal microbiota was clustered in two groups according to the predominant microorganisms; “Enterotype I” was characterized by a predominance of *Ruminococcaceae* family (e.g., *Ruminococcus*), *Lachnospiraceae* family (e.g., *Blautia*) and the genus *Bacteroides and Bifidobacterium* in their gut microbiota, while mothers belonging to the “Enterotype II” were characterized by a dominance of *Prevotella, Peptoniphilus, Anaerococcus*, and *Porphyromonas* genus [28].

The population characteristics with regard to the mother (e.g., birth weight (BW) and weight gain), the gestational aspects (e.g., age, primipara, and antibiotic use) and infant characteristics (i.e., gender, BW, and BMI z-scores) were similar between the two groups of enterotypes (Table 1), allowing us to discard these aspects as confounding factors in the later analysis. However, mothers belonging to Enterotype II displayed a higher frequency of cesarean delivery than the ones belonging to Enterotype I (*p* = 0.027). Moreover, mothers that clustered in Enterotype II had a tendency to be given more antibiotics at delivery and to have a lower gestational age than the cluster of Enterotype I mothers. Besides this, no child showed atopy or intolerance to cow milk protein (data not shown) until month 12. Thus, these variables could not influence our data either.

### 2.2. Analysis of Maternal and Cord Plasma Immune Factors

First, we aimed to characterize the plasma immune component levels of MP and UCP and to study the correlations between them in order to analyze their transplacental transfer. The levels obtained were different in terms of predominance, concentration, and detection. Whereas the most predominant CKs on UCP samples were IL-2, IL-6, and IL-18, the dominant ones in the MP were IL-18, IL-23, and IL-27 (Table 2). Despite the bad detectability of CKs, it was possible to observe differences between the two type of plasma samples. Surprisingly, both IL-1β and TNF-α had a higher concentration and detectability in the UCP than in the MP (*p* < 0.05). Moreover, the IL-2 levels were also higher in the UCP than in the MP. Conversely, other cytokines, such as IFN-γ, IL-9, IL-23, IL-21, and IL-22 were at lower concentrations and had a lower detectability in the UCP with respect to the MP (*p* < 0.05) (Table 2).

As expected, the total Ig concentration was higher in MP than in UCP (*p* < 0.05), which was also consistent with the significantly higher levels of IgM, IgG2, IgA, and IgE (Table 3). In contrast, when comparing the relative percentages of these Ig types and subtypes, the percentages of IgG and IgG1 were higher in the UCP than in the MP (*p* < 0.05), although no differences in the Th1- and Th2-associated Igs were observed (Table 3).

On the other hand, we also analyzed the adipokines (leptin and adiponectin) involved in fetal development and with immunomodulatory actions. Whereas the levels of leptin and the leptin/adiponectin ratio (L/A ratio) were significantly lower in the UCP with respect to the MP, the level of adiponectin was higher in the UCP (*p* < 0.05) (Table 4).

**Figure 1:**
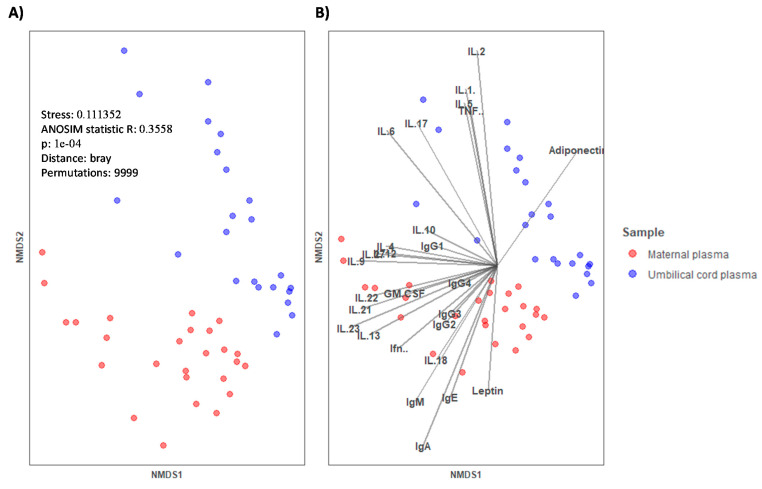
(**A**) Non-metric multi-dimensional scaling (NMDS) for the concentration of immune factors in maternal plasma (MP, *n* = 26) and umbilical cord plasma (UCP, *n* = 23). Categorical variable (type of sample) is represented by color (red for MP and blue for UCP). (**B**) Continuous variables are represented by vectors. Samples were clustered by type of plasma and the analysis of similarities (ANOSIM) test was used to determine if the immune composition was different in the different clusters, *p* = 0.0001. GM-CSF, Granulocyte Macrophage Colony-Stimulating Factor; IFN, Interferon; IL, Interleukin; TNF, tumor necrosis factor. IgG1, IgG2, and IgG3 (Igs associated with Th1 response); IgG4 (Ig associated with Th2 response) [30,31].

In Figure 1, a non-metric multi-dimensional Scaling (NMDS) graph of plasma plotted with the data of immune components (Igs, CKs, and adipokines) is shown. The different clustering of samples can be observed depending on the plasma type (MP in red and UCP in blue). The observation was statistically significant, as assessed by the analysis of similarities (ANOSIM) test for categorical variables (*p* = 0.0001).

### 2.3. Correlations between Immune Factors in the Mother-Infant Pair

**Figure 2:**
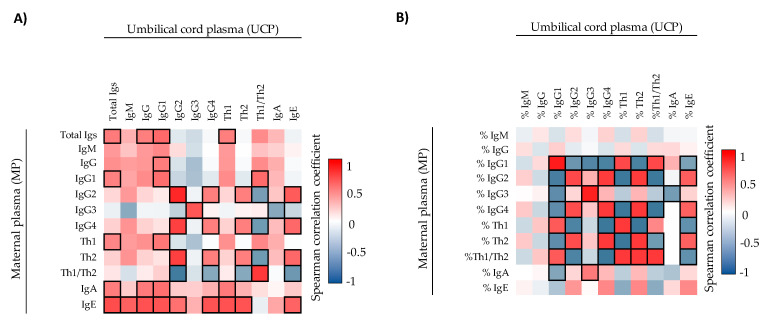
Correlations between the Ig concentrations (**A**) and the Ig relative percentages (**B**) present in umbilical cord plasma and maternal plasma (*n* = 17). The Spearman correlation coefficient is represented in the heat map following the color in the legend. Bold frames represent correlations with statistical significance (*p* < 0.05). MP, maternal plasma; UCP, umbilical cord plasma. IgG1, IgG2, and IgG3 (Igs associated with a Th1 response); IgG4 (Ig associated with a Th2 response) [30,31].

**Figure 3:**
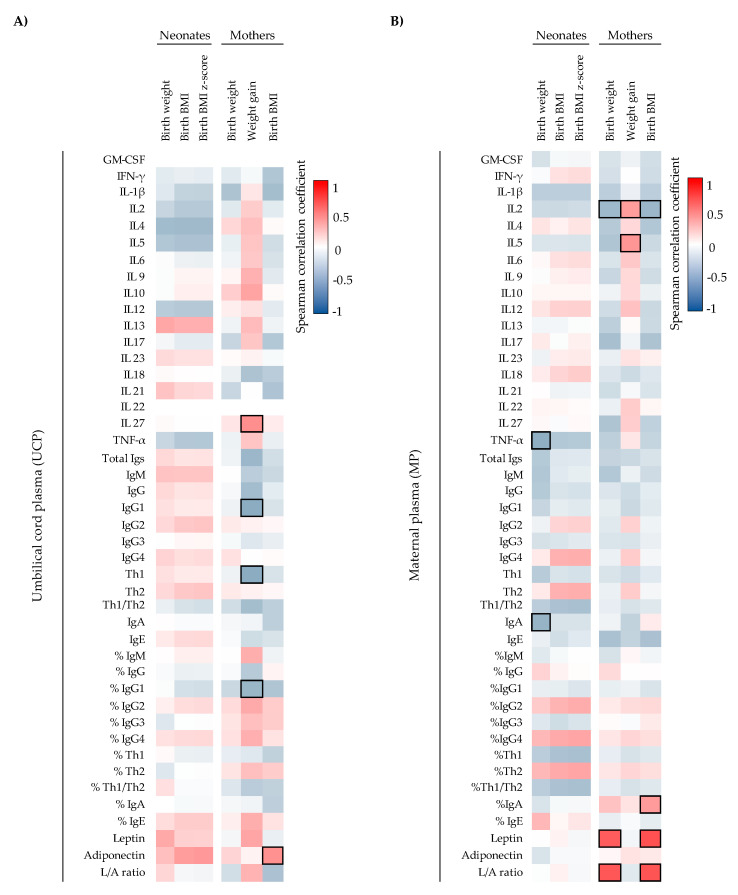
Correlations between CK, Ig, and adipokine composition present in UCP (**A**) (*n* = 23) and MP (**B**) (*n* = 26) with the maternal and infant weight parameters. The Spearman correlation coefficient is represented in the heatmap following the color in the legend. Bold frames represent correlations with statistical significance (*p* < 0.05). MP, maternal plasma; UCP, umbilical cord plasma; GM-CSF, Granulocyte Macrophage Colony-Stimulating Factor; IFN, Interferon; IL, Interleukin; L/A ratio, Leptin/Adiponectin ratio; TNF, tumor necrosis factor. IgG1, IgG2, and IgG3 (Igs associated with Th1 response); IgG4 (Ig associated with Th2 response) [30,31].

Moreover, the correlations between maternal–neonatal pairs in terms of the immune factors quantified were assessed. The initial analysis represented in heatmaps showed that there were many correlations between mother–infant pairs (Figure 2 and Figure 3). Regarding Igs, the concentrations (Figure 2A) and the relative frequencies (Figure 2B) of IgG1, IgG2, IgG3, IgG4, Th2-associated Ig, and Th1/Th2 ratio correlated positively in the mother–infant pair. In contrast, although a correlation with the %Th1-associated Igs was found (Figure 2B), the absolute concentration of Th1-associated Ig response did not correlate (Figure 2A). The overall Igs and the concentration of IgE also correlated positively between MP and UCP. Besides, in general, the correlation coefficients of the relative percentages of Igs were stronger than the absolute values (Figure 2B), such as, for example, %IgG1 (ρ = 0.873, *p* < 0.05), which displayed the highest correlation coefficient between MP and UCP. We only found one significant correlation for CKs: maternal IL-6 was associated positively with infant IL-5 (ρ = 0.513, *p* < 0.05) (Supplementary Figure S2A). The adi-pokine levels in MP and UCP did not display any significant correlation (Supplementary Figure S2B).

To assess the influence of maternal weight on the UCP immune factor levels and the influence of these biocomponents on infant weight, the correlations between these parameters were also studied (Figure 3). A positive correlation between maternal BMI at birth and the adiponectin concentration in UCP (ρ = 0.441, *p* < 0.05) was found (Figure 3A). Additionally, maternal weight gain during the pregnancy was associated positively with IL-27 (ρ = 0.452, *p* < 0.05) and negatively with IgG1 (ρ = -0.473, *p* < 0.05), %IgG1 (ρ = -0.416, *p* < 0.05), and Th1-type Ig (ρ = -0.473, *p* < 0.05) in UCP (Figure 3A). Moreover, the correlations of weight variables with MP immune factors indicated that the higher the concentration of TNF-α and IgA in the mother, the lower the weight of the baby at birth (ρ = -0.459, *p* < 0.05; ρ = -0.433, *p* < 0.05, respectively) (Figure 3B). Besides this, more correlations between maternal weight parameters and their plasma samples were also observed (Figure 3B). For example, the maternal birth weight and maternal birth BMI correlated positively with the maternal leptin levels (ρ = 0.636, *p* < 0.05; ρ = 0.681, *p* < 0.05, respectively) and L/A ratio (ρ = 0.657, *p* < 0.05; ρ = 0.668, *p* < 0.05, respectively) (Figure 3B), contrary to what happened in UCP (Figure 3A).

### 2.4. Influence of Maternal Microbial Enterotype on Plasma Immune Factors

**Figure 4:**
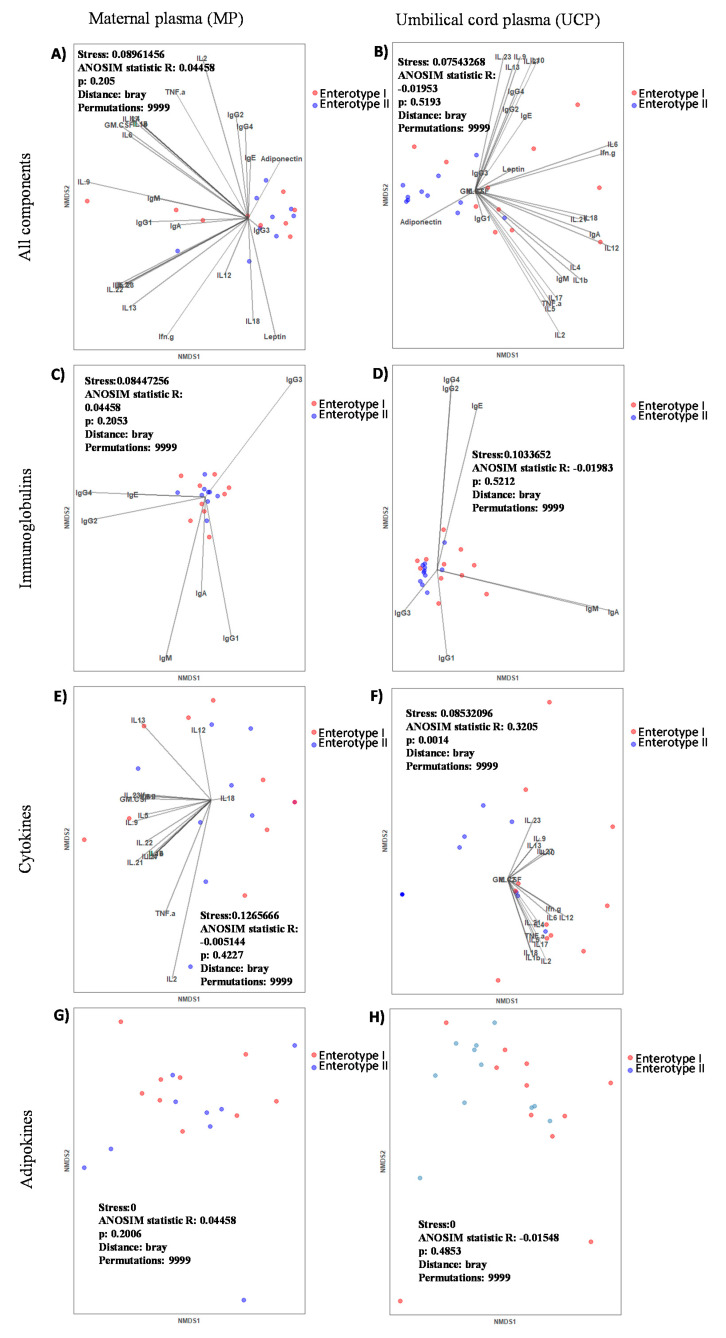
Non-metric multi-dimensional scaling (NMDS) representations of the immune components studied with regard to maternal enterotype in maternal plasma (**A**,**C**,**E**,**G**) and umbilical cord plasma (**B**,**D**,**F**,**H**) showing all components (**A**,**B**): Igs alone (**C**,**D**), CKs alone (**E**,**F**), and adipokines alone (**G**,**H**).

Following the characterization of the immune factors composition present in UCP and MP, the study of the effect of the enterotype on them was assessed. The MP immune factors composition in terms of CKs and Igs did not differ between the two maternal enterotypes (Supplementary Tables S1 and S2). However, an increase in adiponectin concentration in mothers who clustered in the Enterotype II with respect to those belonging to the Enterotype I was observed (Supplementary Table S3). The lack of overall differentiation between enterotypes can be observed in the non-metric multi-dimensional scaling (NMDS) representations in Figure 4. When UCP were grouped by the type of their respective maternal enterotypes (Figure 4B,D,F,H), significant differences in ANOSIM test were observed in the NMDS according to the CK profiles (*p* < 0.05) (Figure 4F). The maternal enterotype though did not originate clearly separated groups in the analysis of the Ig and adipokine profiles (Figure 4B,D,H). Specifically, when analyzing each component, we observed lower UCP values of IgA, %IgA, %IgM, IFN-γ, %IFN-γ, IL-1β, %IL-1β, IL-2, %IL-2, IL-5, IL-6, %IL-6, IL-12, %IL-12, IL-17, TNF-α, and %TNF-α and higher values of %IgG in the Enterotype II group with respect the Enterotype I group (*p* < 0.05) (Table 5 and Table 6). Interestingly, although the UCP adiponectin concentration was not significantly different between clusters (Supplementary Table S3), a great association of adiponectin vector with the aggrupation tendency of UCP from Enterotype II group was observed (Figure 4B).

### 2.5. Influence of Maternal Dietary Components on Maternal and Cord Plasma Immune Factor Composition

**Figure 5:**
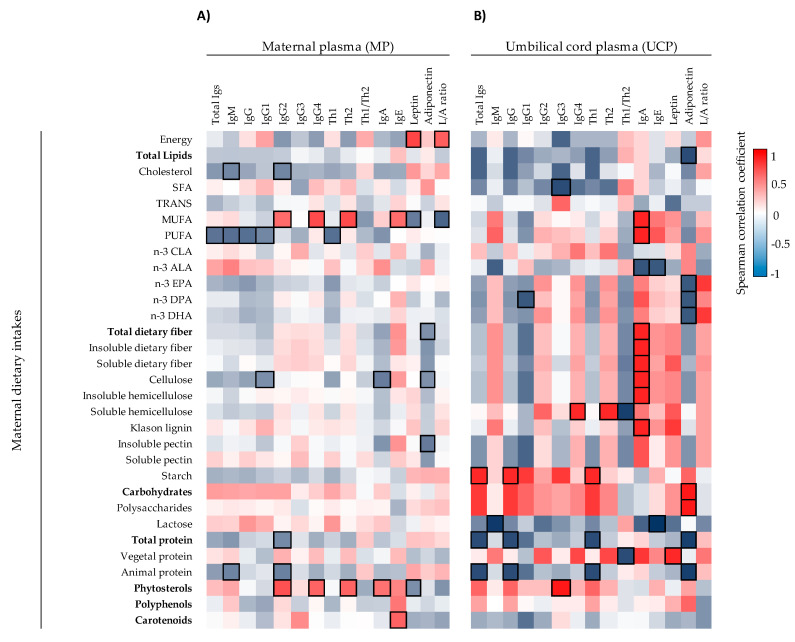
Correlations between Ig and adipokine composition present in MP (**A**) (*n* = 13) and UCP (**B**) (*n* = 6) with the maternal dietary intakes. The Spearman correlation coefficient is represented in the heat map following the color in the legend. Bold frames represent correlations with statistical significance (*p* < 0.05). MP, maternal plasma; UCP, umbilical cord plasma; L/A ratio, Leptin/Adiponectin ratio. IgG1, IgG2 and IgG3 (Igs associated with Th1 response); IgG4 (Ig associated with Th2 response) [30,31].

**Figure 6:**
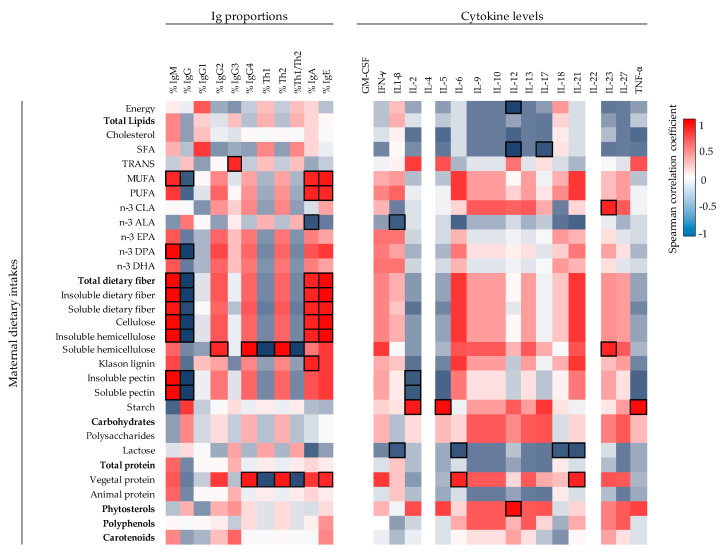
Correlations between Ig proportions, adipokine composition, and CK levels of UCP (*n* = 6) with the maternal dietary intakes. The Spearman correlation coefficient is represented in the heat map following the color in the legend. Bold frames represent correlations with statistical significance (*p* < 0.05). MP, maternal plasma; UCP, umbilical cord plasma; L/A ratio, Leptin/Adiponectin ratio. IgG1, IgG2, and IgG3 (Igs associated with Th1 response); IgG4 (Ig associated with Th2 response) [30,31].

The impact of maternal diet components during the gestation period on the MP and UCP immunological profile was studied in a representative subsample of the cohort of 13 mothers (Figure 5, Figure 6 and Supplementary Figure S3). The Spearman correlation analysis represented in a heat map for each individual dietary component of the diet with the Ig profile in MP (Figure 5A) showed that the dietary intake of monounsaturated fatty acid (MUFA), polyunsaturated fatty acid (PUFA), and phytosterols were those having the maximum impact. Particularly, PUFA correlated inversely to the MP levels of IgM and IgG, the latter due to their influence on IgG1, the predominant Th1-associated Ig. Moreover, the maternal intake of MUFA and phytosterols correlated positively with IgG2 and IgG4 in addition to IgE or IgA, respectively. Regarding CKs, only three significant positive correlations were found: trans-fatty acids with IL-18 and EPA and hemicellulose with IL-21 (Supplementary Figure S3). Furthermore, maternal intake during the gestation of MUFA and phytosterols was associated negatively with leptin levels in MP and associated positively with energy intake (*p* < 0.05). With regard to the other adipokine studied, different fiber types in the maternal diet such as cellulose, total dietary fiber, and pectin correlated negatively with the adiponectin levels (*p* < 0.05) (Figure 5A).

The influence of maternal diet on the immunological profile of the UCP (Figure 5B and Figure 6) was more remarkable than that of the MP. Different components of the maternal diet correlated positively with IgA levels, such as MUFA, PUFA, and many fiber-type components (cellulose, hemicellulose, lignin, and both soluble and insoluble fiber) (*p* < 0.05). In addition, the total and animal protein intake seemed to be associated with lower levels of Ig, particularly those of the Th1 response, whereas starch consumption led to the opposite effect (Figure 5B). Overall, both hemicellulose and vegetal protein consumption during gestation correlated with a lower Th1/Th2 ratio (*p* < 0.05). Moreover, when UCP Igs relative proportions were evaluated, the influence of the intake of MUFA and dietary fiber showed a correlation with lower IgG levels and higher levels of IgA, IgE, and IgM (*p* < 0.05, Figure 6). Stronger associations between CKs and maternal diet were found in UCP with respect to MP (Figure 6). For example, there are significant positive associations between vegetal protein and IL-6, starch with IL-2 and IL-5, phytosterols with IL-12 and CLA, and hemicellulose with IL-23. On the contrary, the maternal intake of SFA was associated negatively to the UCP levels of IL-12 and IL-17 (*p* < 0.05). Finally, the intake of vegetal protein influenced positively the UCP leptin concentration, whereas animal protein, EPA, DPA, and DHA present in the maternal diet were associated negatively with UCP adiponectin concentration (*p* < 0.05) (Figure 5B).

## 3. Discussion

Immune cells and immune factors participate in coordinated communications between the mother and fetus in order to support a successful pregnancy [3,32]. Little is known about the effect of maternal diet and maternal microbial enterotype on the composition of Igs, CKs, and adipokines in UCP. To evaluate this influence, samples from the mother–infant birth cohort in the Spanish–Mediterranean area (MAMI) were used. This knowledge may be used to improve the health and development of the neonate through nutritional interventions in pregnant women.

During pregnancy, the immune system must engage in maintaining tolerance towards the fetus while preserving the immune function and transferring passive immunity to the offspring. Therefore, the network of communication and trafficking of the immune system is pivotal [4]. In this study, we first characterized the immune and adipokine composition of UCP and MP. The overall composition of UCP was clearly different from that of MP. In general, the Ig absolute values were higher in MP than in UCP. However, %IgG and particularly %IgG1 predominated in UCP with respect to MP. These increases seemed to be due to the fact that the other percentages of classes and subclasses were lower in UCP (e.g., %IgM), causing a relative increase even though the changes in the concentrations were not pronounced. Otherwise, these increases could also be due to a real rise in relative proportions because, in addition to the transplacental transfer of IgG from the mother [5,33], the fetus begins to produce its own IgG haplotype from the 10^th^ week of gestation, and this concentration increases considerably on the delivery day [34]. The other classes were found in lower concentrations since, to date, maternal IgG is the only known Ig to cross the placental barrier [33], reaching the fetus by the FcRn receptors expressed by syncytiotrophoblasts [35]. For this reason, IgM, IgA, and IgE are found in very low concentrations in infant circulation [5]. Moreover, not all the IgG subclasses are transferred equally [35]. This fact could explain why we found correlations between MP and UCP in IgG1, IgG2, IgG3, and IgG4 but not in total IgG. The absolute concentration of IgE also showed a correlation between MP and UCP in this study. As IgE does not cross the transplacental barrier [33], other factors may be involved. In this regard, a study found both maternal–fetal and paternal–fetal IgE correlation [36], suggesting that genetics and the home environment could influence the neonatal IgE concentration.

The implantation and parturition in a healthy pregnancy is mediated by pro-inflammatory responses [3], although a tolerant and anti-inflammatory environment is generated during pregnancy [4,10]. A normal-term delivery has been associated with an upregulation of inflammatory CKs such as IL-1β, IL-6, IL-8, and TNF-α [3,11]. It is well known that these CKs are important to lead to correct parturition, such as inducing the contraction of the smooth muscle in the uterus [11,37]. This inflammatory environment at the delivery day could be the reason why we found higher levels of IL-1β and TNF-α in UCP than in the MP. This result is in line with a recent report stablishing that fetal-placental variables are directly associated with changes in the CKs present in UCP [38]. A successful pregnancy needs these pro-inflammatory events, but it is well known that an overexpression of pro-inflammatory CKs can disrupt fetal and placental developmental pathways [3] and can promote pre-term parturition or other delivery disorders [10]. On the other hand, the under-expression of those CKs is associated with adverse pregnancy outcomes. For example, Chehroudi et al. observed lower levels of IL-1β, IL-6, and TNF-α in umbilical cord lysates at birth in mothers suffering from pre-eclampsia and gonococcus infection [38]. They also observed a decrease in IL-10 concentration.

With regard to adipokine levels, we found three-fold higher levels of leptin in MP with respect to UCP, similarly to a study by Schubring et al. [39], in which the leptin levels at 38–40 weeks of gestation were the highest in the entire studied period which corresponded to the first 6 weeks after birth. In contrast, adiponectin was higher in UCP than in MP. Moreover, we did not observe correlations between UCP and MP either for leptin or adiponectin, coinciding with results obtained by other authors [18,39]. Kotani et al. saw that UCP adiponectin concentration was higher than that from normal-weight adults and when the UCP adiponectin concentration was high, the mass of fetal fat also increased [6]. Although we did not observe a correlation between UCP adiponectin and the weight of the neonate at birth, we observed a positive correlation between UCP adiponectin and the maternal BMI at the delivery day, suggesting that an increase in maternal mass fat could increase UCP adiponectin levels. Furthermore, we observed that the higher the maternal weight and BMI, the higher the maternal leptin and L/A ratio, linking with their weight gaining activity, as described previously in literature [40].

Previous studies in the same MAMI cohort showed the impact of specific dietary compounds such as dietary fiber, vegetable protein, polyphenols and lipids (mainly, the n-3 fatty acids DHA and DPA), their enterotype was characterized by predominance of the *Ruminococcaceae* family (e.g., *Ruminococcus*), *Lachnospiraceae* family (e.g., *Blautia*) and the genera *Bacteroides* and *Bifidobacterium*. On the other hand, when they biased towards higher intake of carbohydrates, saturated fatty acids and proteins (mainly animal protein) their enterotype was enriched in the genus *Prevotella*, *Peptoniphilus*, *Anaerococcus*, and *Porphyromonas* [28].

With regard to the influence of the maternal enterotype on MP composition, we can observe that the enterotype had a limited influence; only adiponectin was increased in the Enterotype II group. In contrast, we observed a great impact on UCP composition, suggesting that the gut microbial community of pregnant women affected the Ig, CK and adipokine levels of UCP, which in turn, could have had an impact during pregnancy. Moreover, mothers belonging to Enterotype II group displayed higher frequencies of antibiotic intake at birth and cesarean deliveries. Indeed, we suggest that having Enterotype II increases the susceptibility towards imbalanced immune factors, which could be influenced by the maternal diet and microbiota composition. The most important finding is that CKs, mainly the pro-inflammatory ones (IFN- γ, IL-1β, IL-6, IL-12, IL-17, and TNF-α), were decreased in the Enterotype II group at the day of delivery. These results are in line with the fact that pro-inflammatory CKs play an important role in a normal parturition [3,11,38]. However, it has to be taken into account that the cesarean delivery proportion is higher in this enterotype, and in this situation, high levels of these CKs could not be required. However, the comparison of these factors between the mode of delivery for both the MP and the UCP in our limited number of samples ruled out this possible influence. Nevertheless, it would be interesting to deep into this CKs-enterotype association with higher number of participants, including similar levels of cesarean deliveries in both groups. It has also to be considered the possible influence that the intrapartum antibiotic used in the cesareans would also cause a shift in the maternal microbiota composition.

The Enterotype II group also showed lower levels of leptin and higher levels of adiponectin in MP, despite this change not being significant. In line with this, lower levels of leptin could indicate disorders during pregnancy, because it acts as an hormonal feedback loop that indicates a normal progression of pregnancy [39]. This feedback loop can be done because the syncytiotrophoblast produces adequate amounts of placental leptin which is then secreted into the maternal circulation [41,42]. On the other hand, an increase in adiponectin in both MP and UCP in Enterotype II group was observed. Daryasari et al. observed differences on maternal plasma adiponectin levels depending on the mode of delivery [43]. They found that umbilical cord blood from vaginal delivery had higher levels of adiponectin. However, in our study, we ruled out the possibility that the increase in MP adiponectin of the Enterotype II group was due to the predisposition to have cesarean delivery, because there was not a significant change comparing that component between the types of delivery for both the MP and the UCP.

Besides the maternal microbiota, the dietary components and specific nutrients have also been associated with the neonatal microbiota composition on previous studies with MAMI cohort [27,28]. Because in the present approach a clear association between the immune and adipokine factors and the maternal enterotype was found, the following step was to assess the impact of the maternal diet on immune and adipokine factors on MP and UCP. Surprisingly, more influence of dietary components on the immunological profile of the UCP than MP was found.

It is well established that a diet rich in vegetable protein, fish, fiber, and polyphenols improves healthy conditions both in adults and children [44,45,46,47,48]. Moreover, a diet high in fiber meliorates an anti-inflammatory environment, and protects children from noninfectious colonic diseases and inflammations [44], and improves the treatment of rheumatoid arthritis [45]. The mechanism is still unknown, but fiber intake during pregnancy and lactation could be a key element in improving the infant development and promising health at short and long term. Dietary fiber components (non-digestible carbohydrates) are also broken down and oxidized incompletely by intestinal microbiota forming short-chain fatty acids (SCFA) [49], which can reach the systemic compartment and, in turn, modulate both systemic and mucosal immune functions [50,51,52] and regulate some metabolic aspects [53]. There are evidences that the differentiation of T cells and the expression of IL-10, IL-17, and IFN-γ are supported by intestinal SCFA in mice [54]. It is reported that n-3 PUFA supplementation is needed for pregnant women who do not eat fish, since their deficiency is associated with worse pregnancy outcomes such as low birth weight and preterm delivery [55,56]. Besides, Margherite Maranesi et al. suggested that reproductive performances such as fertility and pregnancy could be enhanced by PUFA supplementation [57]. There is little information available for the influence of maternal dietary components on neonatal immune factors in humans. This is the first study providing associations between maternal dietary intakes of (dietary fiber and other dietary components) with UCP immune factors. Fiber-type components (cellulose, hemicellulose, lignin, and both soluble and insoluble fiber) and MUFA and PUFA correlated positively with UCP IgA. These dietary components are related with the Enterotype I group in which there were higher levels of IgA, higher proportion of IgA and IgM, but less relative percentage of IgG on UCP. Besides this, MUFA and dietary fiber components correlated positively with UCP IgA, IgM, and IgE proportions and negatively with UCP IgG proportion. Overall, the maternal dietary fiber, possibly by raising the production and transplacental transfer of SCFA, seems to have a role in the developing mucosal immune defense of the neonate, although further studies should be performed to test this hypothesis.

Moreover, the maternal intake of lipids influences infant development due to the fatty acids fetal transfer [58,59]. During pregnancy, essential fatty acids (EFAs) are transported actively from the mother across the placenta, since the demand of EFAs (EPA, DHA and ALA) and long-chain polyunsaturated fatty acids (PUFAs) derivatives for fetal development is increased [60,61,62]. Accordingly, our results may indicate that the lipids intake affects the immune and adipokine composition of UCP. As mentioned before, MUFA and PUFA impacted on Ig proportions. However, EFAs, specifically EPA, DPA, and DHA, were correlated negatively with adiponectin levels in UCP. This was also an interesting observation since Enterotype I group was associated with higher intake of DPA and DHA [28] and less UCP adiponectin levels, suggesting that adiponectin could be influenced by EFAs transferred from the mothers, but also this adipokine could be influenced by maternal carbohydrates (polysaccharides), insoluble pectin and animal protein. Although UCP adiponectin has a tendency to decrease in Enterotype I group and the mothers from this group had lower animal protein intake, the association study showed a negative correlation. Another difference between the two enterotype groups was the intake of animal protein [28]. Enterotype I group is characterized by a high intake of vegetal protein. According to the IL-6 increase observed in Enterotype I group, a positive correlation was also observed between vegetal protein and UCP IL-6 concentration, suggesting that the dietary component having an influence on the IL-6 levels in Enterotype I group could be the vegetal protein.

Among all the factors, those that coincide in the enterotype and diet analysis should receive more attention. Although more research elucidating the effect of immune factors on the development and health of the neonate is required, it is of great interest to find that the maternal diet and the maternal enterotype impact more on the levels of the UCP immune and adipokine factors than those of the MP. This is a very important fact since the neonate’s immune system is still developing and changes in these factors could determine the development, the birth, and the health later on. Mechanism of action studies that allow confirm these associations and additional comparative studies may help to provide strong information and new insights into maternal dietary interventions to improve infant development.

Finally, the limitations of this study include sample size, which could have affected the statistical power of the study and also, the low number of matched maternal-neonatal blood and fecal samples. Although the starting number of samples was low for the main objective but enough to obtain statistical differences, the sample dropouts affected the secondary objective outcomes. The details of the sample recruiting can be seen in the flow chart of Supplementary Figure S1, in which can be observed that not all analysis were performed in all subjects. Secondly, the poor CK detectability, which is already described in the literature, also affected the quantitative interpretation of the results, which should be cautiously analyzed. Moreover, some concurring factors such as the Enterotype II and higher number of cesarean deliveries could have had and influence on the results shown here, even though we did not observe a significant effect on the immune components of the umbilical cord plasma. Despite these limitations, our study is a pioneer in providing novel and relevant data on the interactions between diet, microbiota and immunoglobulin and cytokine profiles. Our data provides the base for future studies as the topic warrants further investigation.

## 4. Materials and Methods

### 4.1. Cohort and Study Subjects

A total of 27 mothers and 23 infants were recruited into this study from the MAMI cohort (Supplementary Figure S1). This mother-infant birth cohort from the Spanish-Mediterranean area was set up in the Institute of Agrochemistry and food Technology-National Research Council (IATA-CSIC) in Valencia with the Clinical trial Registry NCT03552939 [29]. All mothers received information about the study, agreed to participate and signed the informed consent. Mothers were required to be older than 18 years, have a healthy pregnancy, be beyond the 37th week of gestation and be able to understand written and spoken Spanish. The exclusion criteria were the use of drugs of medication, the complications during the gestational period or any chronic pathology (such as Diabetes Mellitus type 1 or pre-gestational thyroid problems). Moreover, the area of residence was limited to the Mediterranean countries to ensure lifestyle homogeneity and similar environmental factors among the participants, avoiding the influence on the variables of interest. All the mothers filled out a clinical questionnaire about their health and that of their children, as well as answered other questions of interest for the study (e.g., gestational age, type of delivery and consumption of antibiotics).

Weight and height of the mothers and the infants were measured in medical consultation. Infant growth parameters (BMI z-score) were calculated with the WHO Anthro software (www.who.int/childgrowth/software/en/); accessed on 1 June 2020.

### 4.2. Maternal Plasma and Arterial Umbilical Cord Sampling

Maternal blood was obtained from 27 mothers and collected in sterile containers, coinciding with the collection of blood for other purposes, into anticoagulant-treated tubes (EDTA tubes) immediately prior to delivery (20–30 min before expulsion or before incision in C-section deliveries). Arterial umbilical cord blood was obtained from 23 infants and also collected in EDTA tubes immediately after delivery of the placenta, coinciding with the control blood gases performed on the cord (5 min after birth and immediately after cord clamping). From all the samples, 17 were mother-child pairs and used for correlation studies. All blood samples were sent from hospital to the specimen biobank, and then, managed and processed under specific standardized protocols at “Biobanco para la Investigación Biomédica y en Salud Pública de la Comunidad Valenciana (IBSP-CV)”. In brief, cells were removed from plasma by centrifugation at 1500× *g* for 10 min at 4 °C. The plasma was collected and centrifuged again at 2500× *g* for 10 min at room temperature to deplete platelets in the plasma. The resulting supernatant (clear plasma) was aliquoted and stored at −80 °C until further analysis.

### 4.3. Maternal Microbiota Composition and Enterotype Identification

Maternal fecal microbiota composition at delivery time was studied in some of the participants (Supplementary Figure S1) as detailed previously [28]. Specifically, fecal samples were obtained only from 19 of the 27 mothers involved in the study. Regarding the neonates, samples from their mothers were obtained in most of the cases (22/23). Microbiota composition was assessed by 16S rRNA gene fragment (V3-V4 region) sequencing, as detailed previously [28]. Briefly, Illumina protocols (Nextera XT Index Kit) were performed according to the manufacturer’s instructions (Illumina, Hayward, CA, USA), and PCR libraries were sequenced using a 2 × 300 pb paired-end run (MiSeq Reagent Kit v3) in a MiSeq-Illumina platform (by the FISABIO sequencing service, Valencia, Spain), according to the manufacturer’s instructions (Illumina, Hayward, CA, USA). Obtained reads were searched for residual adaptors using the program Trimmomatic v. 039 [63].

A DADA2 pipeline v. 1.12.1 was used to achieve quality filtering, sequence joining, and chimera removal [64]. After quality examination, the reads were trimmed at the 270th and 210th nucleotide in forward and reverse position, respectively. Using the SILVA v132 database, taxonomic assignment was performed also including the species-level classification. Samples with less than 100 reads were removed from the final study and also those taxa with lower than 3 reads in at least 10% of the total samples number. Microbial counts were transformed to relative abundance for further analysis and taxa that represent less than 0.01% of the total microbial composition were also filtered.

Maternal microbiota clustering was generated at the genus level as described elsewhere [65] using the phyloseq [66], cluster [67], MASS [68], clusterSim [69], and ade4 R packages [70]. The Jensen–Shannon distance and partitioning around medoid (PAM) clustering were used and the optimal number of clusters was calculated by the Calinski-Harabasz (CH) index.

### 4.4. Maternal Nutritional Status Assessment

Dietary records were collected during the first week after birth by a nutritionist using a 140-item Food Frequency Questionnaire (FFQ) in some of the participants (Supplementary Figure S1). Specifically, only 19 maternal dietary records were obtained, being only available 13/27 from the mothers enrolled in the study and in 6/23 from the neonates’ mothers whose UCP was obtained. FFQ information was analyzed for the energy and daily intake of macro- and micronutrients by using the nutrient Food Composition Tables developed by the Centro de Enseñanza Superior de Nutrición Humana y Dietética (CESNID) [71]. The intake of soluble and insoluble fiber types was determined by using the Marlett food composition tables [72]. Polyphenol content was obtained from the Phenol-Explorer [73]. The FFQ data was validated by a 3-day recall food record questionnaire for the intake of dietary nutrients [74].

### 4.5. Determination of Immunoglobulins, Cytokines and Adipokines Concentrations

The quantification of Igs (IgA, IgM, IgE, IgG1, IgG2, IgG3, IgG4), CKs (GM-CSF, IFN-γ, IL-1β, IL-2, IL-4, IL-5, IL-6, IL-9, IL-10, IL-12p70, IL-13, IL-17A, IL-18, IL-21, IL-22, IL-23, IL-27, TNF-α) and adiponectin in MP and UCP was performed by ProcartaPlex^TM^ Multiplex immunoassay (Thermo Fisher Scientific, Vienna, Austria) using an Antibody Isotyping 7-Plex Human ProcartaPlexTM panel, a Th1/Th2/Th9/Th17 Cytokine 18-Plex Human ProcartaPlex^TM^ panel and an Adiponectin Human ProcartaPlex^TM^ Simplex Kit. To develop the technique, the manufacturer instructions were followed as in previous studies [75,76]. Briefly, magnetic microsphere beads labelled with antibodies specific for a single target protein were used. The addition of the beads of interest in the plate leaded to the analysis of multiple targets in a single well. Finally, the plate was run on a Luminex Instrument and analyzed in a ProcartaPlex Analyst Software (MAGPIX^®^ analyzer, Luminex Corporation) at the Flow Cytometry Unit of the Scientific and Technological Centres of the University of Barcelona (CCiT-UB). Assay sensitivity was as follows: 2.11 ng/mL for IgG1; 16.07 ng/mL for IgG2; 0.08 ng/mL for IgG3; 0.56 ng/mL for IgG4; 0.34 ng/mL for IgA; 0.003 ng/mL for IgE; 6.41 ng/mL for IgM; 1.2 pg/mL for GM-CSF; 0.2 pg/mL for IFN-γ; 0.2 pg/mL for IL-1β; 0.8 pg/mL for IL-2; 1.5 pg/mL for IL-4; 0.3 pg/mL for IL-5; 0.4 pg/mL for IL-6; 0.5 pg/mL for IL-9; 0.1 pg/mL for IL-10; 0.04 pg/mL for IL-12p70; 0.1 pg/mL for IL-13; 0.1 pg/mL for IL-17A; 0.4 pg/mL for IL-18; 0.6 pg/mL for IL-21; 8.2 pg/mL for IL-22; 0.9 pg/mL for IL-23; 5.1 pg/mL for IL-27; 0.4 pg/mL for TNF-α; 4.6 pg/mL for adiponectin. One outlier sample was discarded from the cytokine study. As the detectability of cytokines is poor, we also transformed the data into a binary variable (detectable versus undetectable). Moreover, to perform the calculations of the mean of the CKs, we used a 0 pg/mL value for the undetectable samples.

The quantification of leptin was performed by a Quantikine^®^ Colorimetric Sandwich ELISA Kit (R&D Systems, Minneapolis, MN, USA) following the manufacturer instructions. Data were analyzed by Multiskan Ascent v2.6 software (Thermo Fisher Scientific, Vienna, Austria). Assay sensitivity was 7.8 pg/mL.

### 4.6. Statistical Analysis

Data were analyzed in The Statistical Package for the Social Sciences (SPSSv22.0, IBM, Chicago, IL, USA). Results are expressed as mean ± SEM unless otherwise specified. Shapiro-Wilk and Levene’s tests were used to determine normality and homogeneity of data variance, respectively. When variables were not normally distributed, non-parametric tests were used. Spearman correlation coefficient was used to search correlation between variables. Mann-Whitney U tests were used to assess significant differences between groups while chi-square test compared frequencies, such as detectability of CKs. A *p* value < 0.05 was considered significant.

Moreover, clustering of the study groups was analyzed by non-metric multi-dimensional scaling (NMDS) and by the analysis of similarities (ANOSIM) test for categorical variables (*p* < 0.05 was considered significant) in Rstudio using the R package vegan (Community Ecology Package. R package version 2.4-6). In the NMDS plot it was represented the complex dimensional data in 2 dimensions to highlight the similarities between samples in terms of their immune composition. In this term, the further two samples are from each other, the less similar they are. Furthermore, more information was overlayed on ordination NMDS plot with the function “envfit” to represent vectors onto the plot. Longer vectors mean a stronger association with the samples in that direction.

## 5. Conclusions

The results of the present study demonstrated that the maternal enterotype influences the immune and adipokine composition of umbilical cord plasma (UCP) at higher level than that of maternal plasma (MP) and, above all, CKs seem to be the most impacted by the microbiota composition. However, Igs seem to be the factors most influenced when dietary components are analyzed separately.

**Figure 7:**
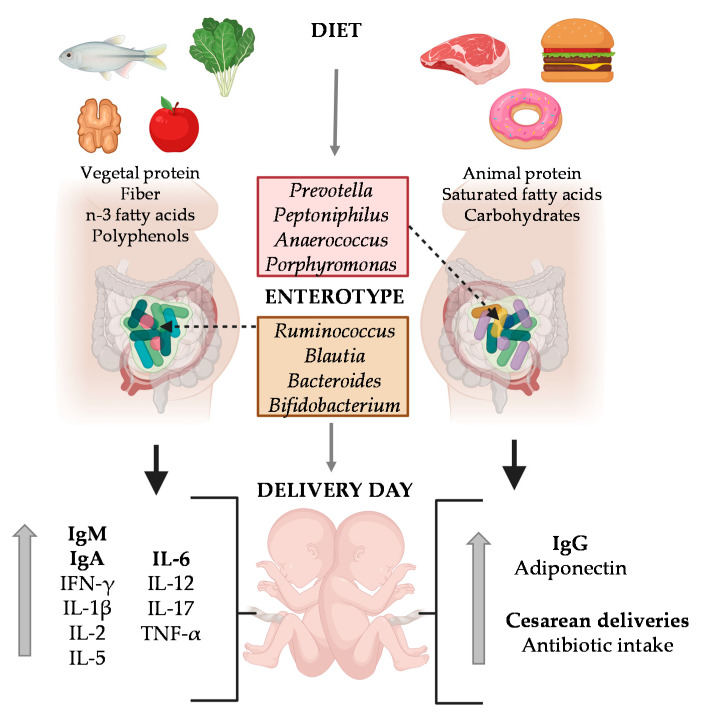
Influence of diet on enterotype, umbilical cord composition and parturition outcomes. The factors marked in bold are the most significant. Created with BioRender.

In summary, it is suggested that a diet rich in fiber, vegetable protein, and n-3 fatty acids with a predominant enterotype in the *Ruminococcaceae* family (e.g., *Ruminococcus*), *Lachnospiraceae* family (e.g., *Blautia*), and the genus *Bacteroides and Bifidobacterium* promotes higher levels of IgA, IgM, and pro-inflammatory cytokines on the delivery day. However, a diet rich in animal protein, carbohydrates, and SFA (with enterotype enriched by the genera *Prevotella, Peptoniphilus, Anaerococcus*, and *Porphyromonas)* generates higher levels of IgG and fewer pro-inflammatory CKs (Figure 7).

Finally, these are interesting findings, and the diet seems to have a high potential to alter the immune composition of umbilical cord plasma. However, there are several perinatal factors, such as mode of delivery, antibiotic exposure, habits, and maternal stress, that could be also influential.

## Figures and Tables

**Table 1:** Mother–infant birth cohort characteristics.

	All	Enterotype I *	Enterotype II *	*p*
**Maternal Characteristics**	***n*** **= 27**	***n*** **= 10**	***n*** **= 9**	
Pre-gestational BMI (Kg/m^2^), ^1^ mean ± SEM	23.61 ± 0.84	23.07 ± 1.33	26.11 ± 1.52	0.149
Pregnancy weight gain (Kg), ^1^ mean ± SEM	11.94 ± 1.00	10.91 ± 1.86	11.24 ± 1.24	0.886
Antibiotic during pregnancy, ^2^ yes (%)	9 (33.33)	5 (50)	2 (22.22)	0.210
Intrapartum antibiotic, ^2^ yes (%)	15 (55.55)	5 (59)	8 (88.89)	0.069
Perinatal antibiotic, ^2^ yes (%)	18 (66.67)	7 (70)	8 (88.89)	0.313
Gestational age (weeks), ^1^ mean ± SEM	38.78 ± 0.26	38.70 ± 0.40	37.78 ± 0.22	0.065
Mode of delivery: vaginal birth, ^2^ yes (%)	13 (48.15)	6 (60)	1 (11.11)	0.027 *
Primipara, ^2^ yes (%)	11 (40.74)	7 (70)	4 (44.44)	0.260
Gestational diabetes mellitus (GDM), ^2^ yes (%)	3 (11.11)	0 (0)	2 (22.22)	0.115
**Infant Characteristics**	***n*** **= 23**	***n*** **= 11**	***n*** **= 11**	
Gender: Female, ^2^ yes (%)	6 (26.09)	2 (18.18)	4 (36.36)	0.252
Birth weight (kg), ^1^ mean ± SEM	3.04 ± 0.12	3.14 ± 0.21	2.99 ± 0.15	0.915
BMI z-score at birth, ^1^ mean ± SEM	−0.70 ± 0.28	−0.69 ± 0.39	−0.52 ± 0.39	0.575
1st month	−0.75 ± 0.28	−0.60 ± 0.44	−0.84 ± 0.41	0.064
6th month	−0.27 ± 0.17	−0.16 ± 0.32	−0.40 ± 0.16	1.126
12th month	0.11 ± 0.17	0.17 ± 0.19	0.07 ± 0.30	0.373

Maternal (*n* = 27) and infant (*n* = 23) characteristics associated with maternal and umbilical cord plasma samples (MP and UCP, respectively). Subjects were classified in Enterotype I or Enterotype II groups depending on the maternal gut microbial enterotype. Enterotype was determined in selected samples (*n* = 19–22). ^1^ *p* values were calculated with Student’s t-test. ^2^ *p* values were calculated using a X^2^ test. BMI, body mass index. S.E.M: standard error of the mean. * Enterotype group was obtained by using samples with available data on the maternal microbiota (see Materials and Methods Section).

**Table 2:** Detectability and concentrations of cytokines in maternal and umbilical cord plasma.

Cytokines	MP, *n* = 26			UCP, *n* = 23		
	pg/mL	%det	IQR	pg/mL	%det	IQR
**GM-CSF**	1.83 ± 0.98	15.38 (4/26)	0.00–0.00	0.00 ± 0.00	0.00 (0/23) *	0.00–0.00
**IFN-γ**	20.17 ± 4.36	96.15 (25/26)	7.02–26.43	1.80 ± 0.67 *	39.13 (9/23) *	0.00–2.12
**IL-1β**	0.08 ± 0.08	3.85 (1/26)	0.00–0.00	1.43 ± 0.56 *	56.52 (13/23) *	0.00–2.10
**IL-2**	1.62 ± 0.60	26.92 (7/26)	0.00–4.51	9.27 ± 2.66 *	52.17 (12/23)	0.00–18.14
**IL-4**	17.58 ± 9.84	23.08 (6/26)	0.00–6.50	0.53 ± 0.37	8.70 (2/23)	0.00–0.00
**IL-5**	0.67 ± 0.36	42.31 (11/26)	0.00–0.10	6.24 ± 3.30	56.52 (13/23)	0.00–9.09
**IL-6**	10.57 ± 3.57	46.15 (12/26)	0.00–12.42	13.72 ± 7.05	17.39 (4/23) *	0.00–0.00
**IL-9**	5.60 ± 2.29	42.31 (11/26)	0.00–9.46	0.95 ± 0.72 *	17.39 (4/23) *	0.00–0.00
**IL-10**	0.07 ± 0.07	7.69 (2/26)	0.00–0.00	0.06 ± 0.04	13.04 (3/23)	0.00–0.00
**IL-12**	0.31 ± 0.10	65.38 (17/26)	0.00–0.44	0.15 ± 0.03	82.61 (19/23)	0.05–0.24
**IL-13**	3.34 ± 1.09	38.46 (10/26)	0.00–5.10	0.15 ± 0.15 *	4.35 (1/23) *	0.00–0.00
**IL-17**	3.46 ± 2.24	15.38 (4/26)	0.00–0.00	4.88 ± 1.84	30.43 (7/23)	0.00–12.16
**IL-18**	39.95 ± 5.81	100.00 (26/26)	24.89–44.14	12.64 ± 2.40 *	95.65 (22/23)	4.53–16.96
**IL-21**	5.57 ± 1.71	50.00 (13/26)	0.00–10.25	0.58 ± 0.39 *	13.04 (3/23) *	0.00–0.00
**IL-22**	2.86 ± 1.15	23.08 (6/26)	0.00–1.75	0.00 ± 0.00 *	0.00 (0/23) *	0.00–0.00
**IL-23**	32.14 ± 7.87	80.77 (21/26)	0.54–62.86	5.78 ± 4.17 *	17.39 (4/23) *	0.00–0.00
**IL-27**	25.59 ± 12.76	26.92 (7/26)	0.00–13.36	0.81 ± 0.62	8.70 (2/23)	0.00–0.00
**TNF-α**	0.03 ± 0.02	11.54 (3/26)	0.00–0.00	1.16 ± 0.65 *	52.17 (12/23) *	0.00–0.99

Data shown are expressed as mean ± S.E.M., detectability frequencies (%det), and interquartile ranges (IQRs). Mann–Whitney U test was used to determine significant differences between plasma samples. X2 test compared detectability. * *p* < 0.05. MP, maternal plasma; UCP, umbilical cord plasma; GM-CSF, Granulocyte Macrophage Colony-Stimulating Factor; IFN, Interferon; IL, Interleukin; TNF, tumor necrosis factor.

**Table 3:** Concentrations and relative frequencies of immunoglobulins in maternal and umbilical cord plasma.

Immunoglobulins		MP, *n* = 27		UCP, *n* = 23	
		mg/L	%	mg/L	%
**Total**		11,261.98 ± 992.16	-	7524.38 ± 607.52 *	-
**IgM**		3151.52 ± 438.11	27.02 ± 1.92	671.94 ± 54.04 *	9.66 ± 0.79 *
**IgG**		7703.94 ± 722.07	69.08 ± 1.97	6829.39 ± 595.40	90.02 ± 0.90 *
	**IgG1**	5911.40 ± 709.55	73.94 ± 1.71	5452.28 ± 532.65	78.63 ± 1.35 *
	**IgG2**	778.52 ± 60.11	11.36 ± 0.99	541.50 ± 70.35 *	8.18 ± 0.70 *
	**IgG3**	892.46 ± 70.90	12.90 ± 1.17	737.07 ± 53.50	11.68 ± 0.87
	**IgG4**	121.56 ± 12.55	1.79 ± 0.20	98.54 ± 14.42	1.51 ± 0.17
	**Th1**	7582.38 ± 721.55	98.21 ± 0.20	6730.84 ± 589.38	98.49 ± 0.17
	**Th2**	121.56 ± 12.55	1.79 ± 0.20	98.54 ± 14.42	1.51 ± 0.17
	**Th1/Th2**	77.31 ± 10.24	--	82.68 ± 7.98	--
**IgA**		405.77 ± 28.07	3.89 ± 0.22	22.68 ± 9.00 *	0.32 ± 0.14 *
**IgE**		0.75 ± 0.06	0.0074 ± 0.0006	0.37 ± 0.03 *	0.0051 ± 0.0003 *

Data shown are expressed as mean ± S.E.M. and relative frequencies (%). Mann–Whitney U test was used to determine significant differences between plasma samples. * *p* < 0.05. MP, maternal plasma; UCP, umbilical cord plasma. IgG1, IgG2, and IgG3 (Igs associated with Th1 response); IgG4 (Ig associated with Th2 response) [30,31].

**Table 4:** Adipokine levels in maternal and umbilical cord plasma.

Adipokines	MP, *n*= 27	UCP, *n* = 23
	ng/mL	ng/mL
**Leptin**	22.62 ± 4.15	8.08 ± 1.10 *
**Adiponectin**	13,456.27 ± 824.93	29,938.34 ± 3101.71 *
**L/A ratio**	1.85 × 10^−3^ ± 3.82 × 10^−4^	3.31 × 10^−4^ ± 5.95 × 10^−5^ *

Data shown are expressed as mean ± S.E.M. Mann–Whitney U test was used to determine significant differences between plasma samples. * *p* < 0.05. MP, maternal plasma; UCP, umbilical cord plasma. L/A ratio, Leptin/Adiponectin ratio.

**Table 5:** Immunoglobulin composition of umbilical cord plasma from each maternal enterotype.

Immunoglobulins		Enterotype I, *n* = 11		Enterotype II, *n* = 11	
		pg/mL	%	pg/mL	%
**Total**		7591.44 ± 1011.25	-	7746.90 ± 759.91	-
**IgM**		762.62 ± 99.96	11.14 ± 1.48	601.00 ± 41.08	8.10 ± 0.48 *
**IgG**		6786.45 ± 1007.05	88.23 ± 1.69	7140.09 ± 726.53	91.85 ± 0.47 *
	**IgG1**	5386.84 ± 920.83	77.52 ± 2.41	5722.82 ± 634.95	79.41 ± 1.51
	**IgG2**	572.12 ± 123.57	8.94 ± 1.28	529.48 ± 83.70	7.38 ± 0.71
	**IgG3**	715.23 ± 73.33	11.74 ± 1.32	799.95 ± 74.10	12.01 ± 1.26
	**IgG4**	112.25 ± 24.92	1.80 ± 0.32	87.85 ± 17.17	1.20 ± 0.14
	**Th1**	6674.20 ± 999.90	98.20 ± 0.32	7052.24 ± 714.40	98.80 ± 0.14
	**Th2**	112.25 ± 24.92	1.80 ± 0.32	87.85 ± 17.17	1.20 ± 0.14
	**Th1/Th2**	73.19 ± 11.83	-	94.37 ± 11.17	-
**IgA**		41.98 ± 16.97	0.62 ± 0.26	5.44 ± 3.65 *	0.05 ± 0.03 *
**IgE**		0.38 ± 0.05	0.0054 ± 0.0005	0.36 ± 0.04	0.0048 ± 0.0003

Immunoglobulin composition in each enterotype is expressed as mean ± S.E.M. and relative frequencies (%). Mann–Whitney U tests were used to determine significant differences between groups of maternal enterotypes. * *p* < 0.05. IgG1, IgG2, and IgG3 (Igs associated with Th1 response); IgG4 (Igs associated with Th2 response) [30,31].

**Table 6:** Cytokine composition of umbilical cord plasma from each maternal enterotype.

Cytokines	Enterotype I, *n* = 11			Enterotype II, *n* = 11		
	pg/mL	%det	IQR	pg/mL	%det	IQR
**GM-CSF**	0.00 ± 0.00	0.00 (0/11)	0.00–0.00	0.00 ± 0.00	0.00 (0/11)	0.00–0.00
**IFN- γ**	3.12 ± 1.23	63.64 (7/11)	0.00–4.96	0.64 ± 0.47 *	18.18 (2/11) *	0.00–0.00
**IL-1β**	2.44 ± 1.06	81.82 (9/11)	0.29–2.00	0.24 ± 0.19 *	27.27 (3/11) *	0.00–0.29
**IL-2**	14.04 ± 4.76	72.73 (8/11)	0.00–21.94	3.70 ± 1.98 *	27.27 (3/11) *	0.00–11.28
**IL-4**	1.12 ± 0.75	18.18 (2/11)	0.00–0.00	0.00 ± 0.00	0.00 (0/11)	0.00–0.00
**IL-5**	11.14 ± 6.62	72.73 (8/11)	0.00–13.90	0.85 ± 0.82 *	36.36 (4/11)	0.00–0.10
**IL-6**	28.70 ± 13.62	36.36 (4/11)	0.00–73.35	0.00 ± 0.00 *	0.00 (0/11) *	0.00–0.00
**IL-9**	1.88 ± 1.49	27.27 (3/11)	0.00–1.13	0.10 ± 0.10	9.09 (1/11)	0.00–0.00
**IL-10**	0.12 ± 0.09	18.18 (2/11)	0.00–0.00	0.01 ± 0.01	9.09 (1/11)	0.00–0.00
**IL-12**	0.22 ± 0.05	100 (11/11)	0.14–0.33	0.09 ± 0.03 *	63.64 (7/11) *	0.00–0.14
**IL-13**	0.32 ± 0.32	9.09 (1/11)	0.00–0.00	0.00 ± 0.00	0.00 (0/11)	0.00–0.00
**IL-17**	8.02 ± 3.31	45.45 (5/11)	0.00–12.16	0.57 ± 0.57 *	9.09 (1/11)	0.00–0.00
**IL-18**	16.86 ± 4.27	90.91 (10/11)	8.36–23.34	8.98 ± 2.18	100 (11/11)	4.53–11.92
**IL-21**	1.20 ± 0.79	27.27 (3/11)	0.00–1.75	0.00 ± 0.00	0.00 (0/11)	0.00–0.00
**IL-22**	0.00 ± 0.00	0.00 (0/11)	0.00–0.00	0.00 ± 0.00	0.00 (0/11)	0.00–0.00
**IL-23**	11.30 ± 8.57	27.27 (3/11)	0.00–8.64	0.79 ± 0.79	9.09 (1/11)	0.00–0.00
**IL-27**	1.70 ± 1.28	18.18 (2/11)	0.00–0.00	0.00 ± 0.00	0.00 (0/11)	0.00–0.00
**TNF-α**	2.12 ± 1.32	72.73 (8/11)	0.00–2.09	0.15 ± 0.09 *	27.27 (3/11) *	0.00–0.18

Cytokine composition in each enterotype is expressed as mean ± S.E.M., percentage of detectability (%det), and interquartile range (IQR). Mann–Whitney U tests were used to determine significant differences between plasma samples. X2 test compared detectability. * *p* < 0.05. GM-CSF, Granulocyte Macrophage Colony-Stimulating Factor; IFN, Interferon; IL, Interleukin; TNF, tumor necrosis factor.

## Data Availability

Not applicable.

## Supplementary Materials

Supplementary Materials can be found at https://www.mdpi.com/1422-0067/22/4/1778/s1.

## Abbreviations

ALA	Alpha-lipoic acid
ANOSIM	Analysis of similarities
BMI	Body mass index
CLA	Conjugated linoleic acid
CK	Cytokine
DHA	Docosahexaenoic
DPA	Docosapentaenoic acid
EFAs	Essential fatty acids
EPA	Eicosapentaenoic
GM-CSF	Granulocyte macrophage colony-stimulating factor
IFN	Interferon
Ig	Immunoglobulin
IL	Interleukin
L/A ratio	Leptin/Adiponectin ratio
MP	Maternal plasma
MUFA	Monounsaturated fatty acid
NMDS	Non-metric multi-dimensional scaling
PUFA	Polyunsaturated fatty acid
SEM	Standard error media
SFA	Satturated fatty acid
TNF	Tumor necrosis factor
UCP	Umbilical cord plasma
