# Supplementary material for: Association of Maternal Microbiota and Diet in Cord Blood Cytokine and Immunoglobulin Profiles
Source: Int J Mol Sci. 2021 Feb 10;22(4):1778. doi: 10.3390/ijms22041778 (PMC7916816; doi:10.3390/ijms22041778)
Supplement: Supplementary file 1 [file ijms-22-01778-s001.pdf]

Supplementary Table S1. Immunoglobulin composition on maternal plasma (MP) regarding the maternal enterotype.

Immunoglobulins		Enterotype I, n = 10		Enterotype II, n = 9	
		**mg/L**	**%**	**mg/L**	**%**
**Total**		13207.95 ± 2350.97	-	9683.06 ± 812.32	-
**IgM**		3968.92 ± 1105.15	27.84 ± 4.56	2728.42 ± 322.15	27.87 ± 1.90
**IgG**		8800.27 ± 1685.73	68.46 ± 4.55	6530.36 ± 535.74	67.70 ± 2.05
	**IgG1**	7007.03 ± 1686.61	75.03 ± 3.40	4750.65 ± 496.19	71.71 ± 2.78
	**IgG2**	812.50 ± 106.30	11.29 ± 2.13	797.94 ± 127.92	12.32 ± 1.45
	**IgG3**	850.29 ± 109.88	11.82 ± 1.99	856.04 ± 100.30	14.04 ± 2.03
	**IgG4**	130.44 ± 21.77	1.86 ± 0.44	125.73 ± 27.46	1.92 ± 0.30
	**Th1**	8669.83 ± 1686.47	98.14 ± 0.44	6404.64 ± 522.75	98.08 ± 0.30
	**Th2**	130.44 ± 21.77	1.86 ± 0.44	125.73 ± 27.46	1.92 ± 0.30
	**Th1/Th2**	84.78 ± 19.36	-	62.53 ± 10.29	-
**IgA**		437.98 ± 49.81	3.69 ± 0.33	423.48 ± 36.77	4.43 ± 0.28
**IgE**		0.78 ± 0.07	0.0069 ± 0.0009	0.79 ± 0.12	0.0083 ± 0.0011

Data shown are immunoglobulin concentrations expressed as mean ± SEM and relative frequencies (%). Mann-Whitney U test was used to determine significant differences. *$p < 0.05$. MP, maternal plasma; UCP, umbilical cord plasma. IgG1, IgG2 and IgG3 (Igs-associated Th1 response); IgG4 (Ig-associated Th2 response).

Supplementary table S2. Cytokine composition on maternal plasma (MP) regarding the maternal enterotype.

Cytokines	Enterotype I, n = 9		Enterotype II, n = 9	
	**pg/mL**	**%det**	**pg/mL**	**%det**
**GM-CSF**	4.56 ± 2.46	33.33 (3/9)	0.00 ± 0.00	0.00 (0/0)
**IFN- γ**	13.31 ± 4.20	100 (9/9)	15.11 ± 3.26	100 (9/9)
**IL-1β**	0.23 ± 0.22	11.11 (1/9)	0.00 ± 0.00	0.00 (0/9)
**IL-2**	2.26 ± 1.24	33.33 (3/9)	1.43 ± 1.00	22.22 (2/9)
**IL-4**	28.54 ± 24.14	22.22 (2/9)	0.00 ± 0.00	0.00 (0/9)
**IL-5**	0.74 ± 0.68	33.33 (3/9)	0.75 ± 0.72	44.44 (4/9)
**IL-6**	8.26 ± 5.08	44.44 (4/9)	1.56 ± 1.36	33.33 (3/9)
**IL-9**	4.75 ± 3.06	44.44 (4/9)	1.43 ± 1.30	22.22 (2/9)
**IL-10**	0.02 ± 0.02	11.11 (1/9)	0.00 ± 0.00	0.00 (0/9)
**IL-12**	0.18 ± 0.07	55.56 (5/9)	0.31 ± 0.21	55.56 (5/9)
**IL-13**	4.09 ± 1.92	44.44 (4/9)	2.20 ± 2.20	11.11 (1/9)
**IL-17**	2.59 ± 2.45	11.11 (1/9)	0.00 ± 0.00	0.00 (0/9)
**IL-18**	30.01 ± 5.57	100 (9/9)	37.62 ± 4.24	100 (9/9)
**IL-21**	4.28 ± 1.91	44.44 (4/9)	3.84 ± 1.65	55.56 (5/9)
**IL-22**	2.20 ± 2.09	11.11 (1/9)	1.77 ± 1.18	22.22 (2/9)
**IL-23**	22.65 ± 10.00	77.78 (7/9)	23.62 ± 9.96	88.89 (8/9)
**IL-27**	32.38 ± 26.52	22.22 (2/9)	0.58 ± 0.58	11.11 (1/9)
**TNF-α**	0.05 ± 0.05	11.11 (1/9)	0.02 ± 0.02	11.11 (1/9)

Data shown are cytokine concentrations (pg/mL) expressed as mean ± S.E.M. and detectability frequencies (%det). Mann-Whitney U test was used to determine significant differences between plasma samples. Chi-square test compared detectability. * $p < 0.05$. MP, maternal plasma; UCP, umbilical cord plasma; GM-CSF, Granulocyte Macrophage Colony-Stimulating Factor; IFN, Interferon; IL, Interleukin; TNF, tumor necrosis factor.

Supplementary Table S3. Adipokine composition on maternal plasma (MP) and umbilical cord plasma (UCP) regarding the maternal enterotype.

MP	Enterotype I, n = 10 (ng/mL)	Enterotype II, n = 9 (ng/mL)
**Leptin**	21.94 ± 6.71	31.40 ± 7.90
**Adiponectin**	11047.38 ± 1138.75	15605.91 ± 1461.99 *
**Ratio L/A**	2.00E-03 ± 5.81E-04	2.38E-03 ± 8.39E-04

UCP	Enterotype I, n = 11 (ng/mL)	Enterotype II, n = 11 (ng/mL)
**Leptin**	9.40 ± 1.66	6.88 ± 1.59
**Adiponectin**	26124.67 ± 3656.35	34568.60 ± 5153.06
**Ratio L/A**	4.49E-04 ± 1.09E-04	2.14E-04 ± 4.11E-05

Data shown are adipokine concentrations (ng/mL) expressed as mean ± S.E.M. Mann-Whitney U tests were used to determine significant differences between plasma samples. *$p < 0.05$. L/A ratio, leptin/adiponectin ratio.

Supplementary Figure S1

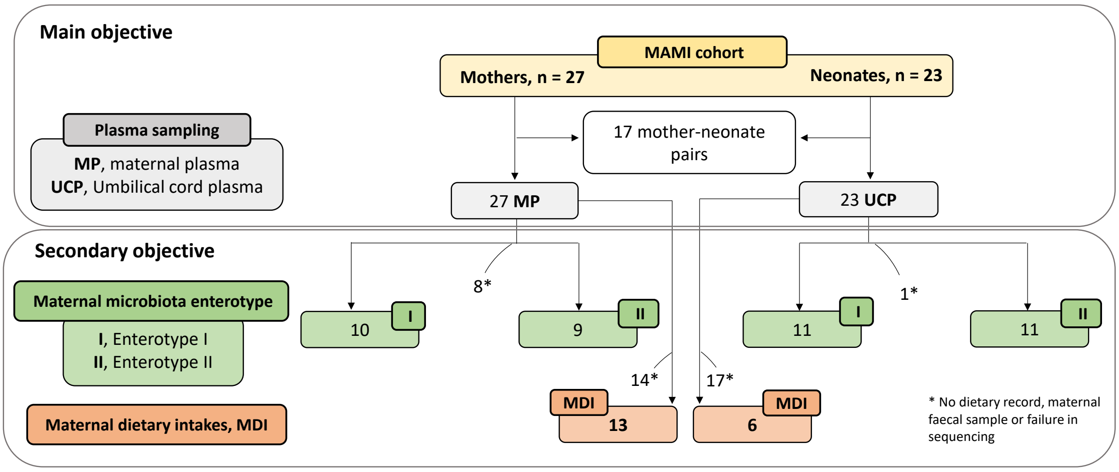

Supplementary Figure S1. Flow chart of the participants and samples available in this study.

Supplementary Figure S2.

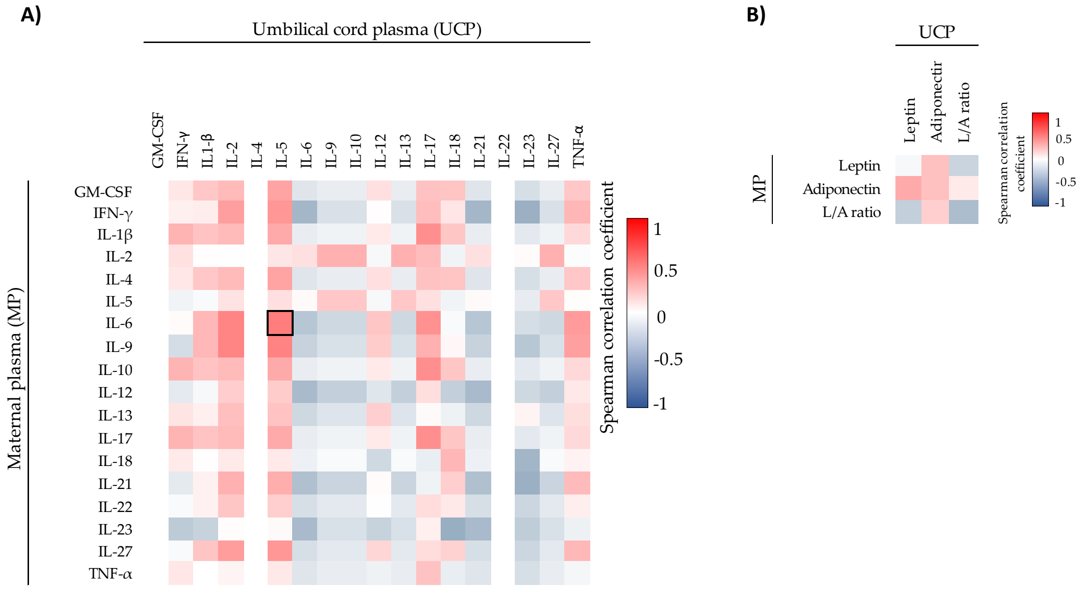

Supplementary figure S2. Correlations between the CK (**A**) and adipokine (**B**) concentrations present in maternal plasma (MP) and umbilical cord plasma (UCP) (n = 16). The Spearman correlation coefficient is represented in the heat map following the color in the legend. Bold frames represent correlations with statistical significance ($p < 0.05$). GM-CSF, Granulocyte Macrophage Colony-Stimulating Factor; IFN, Interferon; IL, Interleukin; L/A ratio, Leptin/Adiponectin ratio; TNF, tumour necrosis factor.

Supplementary Figure S3.

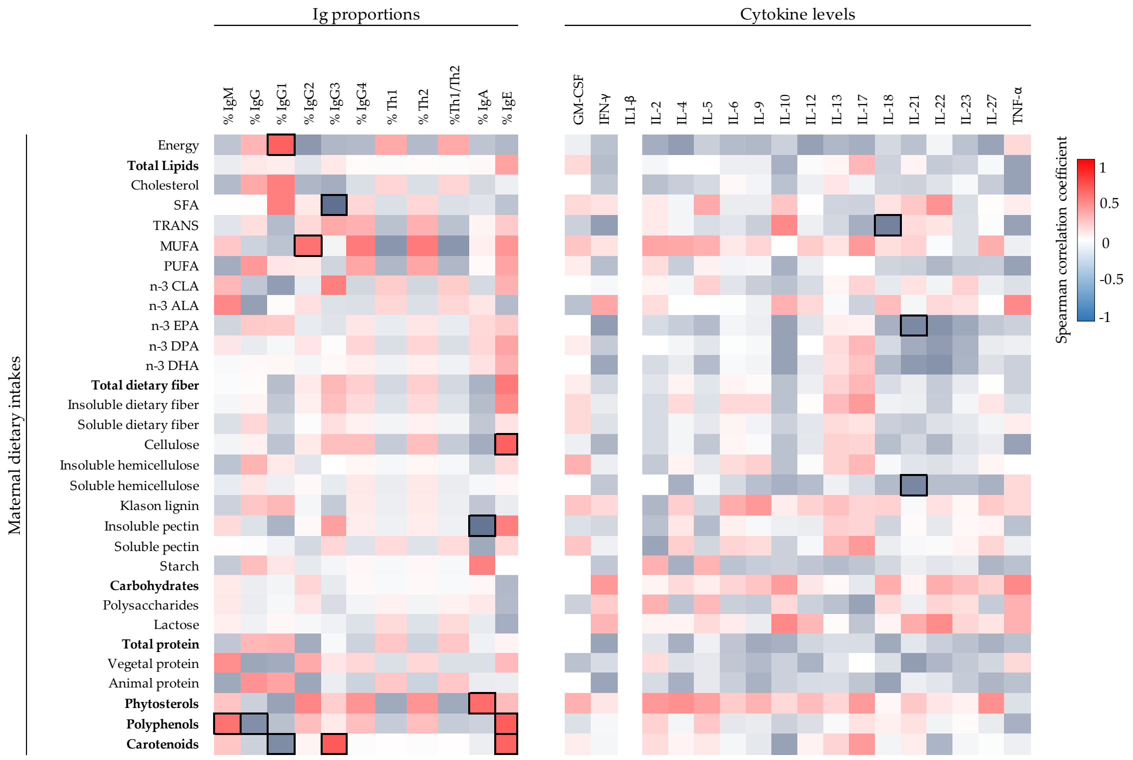

Supplementary Figure S3. Correlations between Ig proportions and CK levels of MP (n = 13) with the maternal dietary intakes. The Spearman correlation coefficient is represented in the heat map following the color in the legend. Bold frames represent correlations with statistical significance (*p* < 0.05). L/A ratio, Leptin/Adiponectin ratio. IgG1, IgG2 and IgG3 (Igs associated to Th1 response); IgG4 (Ig associated to Th2 response).
